# Efficacy of Lipid-Lowering Therapy during Cardiac Rehabilitation in Patients with Diabetes Mellitus and Coronary Heart Disease

**DOI:** 10.3390/jcdd8090105

**Published:** 2021-08-30

**Authors:** Thomas Wittlinger, Bernhard Schwaab, Heinz Völler, Christa Bongarth, Viktoria Heinze, Kristina Eckrich, Manju Guha, Michael Richter, Axel Schlitt

**Affiliations:** 1 Asklepios Harzkliniken GmbH, Kösliner Strasse 12, 38642 Goslar, Germany; 2 Medizinische Fakultät, Universität zu Lübeck, D-23562 Lübeck, Germany; prof.schwaab@drguth.de; 3 Department of Rehabilitation Medicine, Faculty of Health Sciences Brandenburg, University of Potsdam, D-14469 Potsdam, Germany; Heinz.Voeller@klinikamsee.com; 4 Klinik am See, Department of Cardiology, 15562 Rüdersdorf, Germany; 5 Clinic Höhenried, 82347 Bernried, Germany; christa.bongarth@hoehenried.de; 6 Paracelsus-Harz-Clinic, 06485 Bad Suderode, Germany; viktoria.heinze@pkd.de (V.H.); axel.schlitt@pkd.de (A.S.); 7 Clinic Tharandter Wald—Hetzdorf, 09633 Halsbrücke, Germany; kemange@gmx.de; 8 Rehabilitation Clinic Sendesaal, 28329 Bremen, Germany; dr.guha@rehaklinik-sendesaal.de; 9 Coordination Center for Clinical Studies, Martin Luther-University Halle Wittenberg, 06120 Halle, Germany; richter.michael@kks-halle.de; 10 Medical Faculty, Martin Luther-University, 06120 Halle Wittenberg, Germany

**Keywords:** coronary heart disease, cardiac rehabilitation, diabetes mellitus, lipid-lowering therapy

## Abstract

Background: Cardiac rehabilitation (CR) in patients with coronary heart disease (CHD) increases adherence to a healthy lifestyle and to secondary preventive medication. A notable example of such medication is lipid-lowering therapy (LLT). LLT during CR improves quality of life and prognosis, and thus is particularly relevant for patients with diabetes mellitus, which is a major risk factor for CHD. Design: A prospective, multicenter registry study with patients from six rehabilitation centers in Germany. Methods: During CR, 1100 patients with a minimum age of 18 years and CHD documented by coronary angiography were included in a LLT registry. Results: In 369 patients (33.9%), diabetes mellitus was diagnosed. Diabetic patients were older (65.5 ± 9.0 vs. 62.2 ± 10.9 years, *p* < 0.001) than nondiabetic patients and were more likely to be obese (BMI: 30.2 ± 5.2 kg/m^2^ vs. 27.8 ± 4.2 kg/m^2^, *p* < 0.001). Analysis indicated that diabetic patients were more likely to show LDL cholesterol levels below 55 mg/dL than patients without diabetes at the start of CR (Odds Ratio (OR) 1.9; 95% CI 1.3 to 2.9) until 3 months of follow-up (OR 1.9; 95% CI 1.2 to 2.9). During 12 months of follow-up, overall and LDL cholesterol levels decreased within the first 3 months and remained at the lower level thereafter (*p* < 0.001), irrespective of prevalent diabetes. At the end of the follow-up period, LDL cholesterol did not differ significantly between patients with or without diabetes mellitus (*p* = 0.413). Conclusion: Within 3 months after CR, total and LDL cholesterol were significantly reduced, irrespective of prevalent diabetes mellitus. In addition, CHD patients with diabetes responded faster to LTT than nondiabetic patients, suggesting that diabetic patients benefit more from LLT treatment during CR.

## 1. Introduction

Diabetes mellitus constitutes a major risk factor for developing coronary heart disease (CHD), and potentiates the risk for fatal events in patients who already have CHD [1]. Standard treatment of CHD in patients with and without diabetes typically comprises a combination of lifestyle changes, e.g., physical activity on a regular basis, cessation of smoking, adoption of a healthier diet, and secondary preventive medication. The pharmacotherapy includes angiotensin-receptor blockers, ACE inhibitors, beta-blocking agents, and platelet inhibitors. A major goal in the treatment of diabetes mellitus is the reduction of blood lipid levels [2]. Here, lipid-lowering drugs represent one of the most important therapeutic interventions [3].

Currently, the medical consensus recommends lipid-lowering therapy (LLT) for all patients who have developed CHD, irrespective of whether they have diabetes [4,5]. This treatment is independent of the initial level of low-density lipoprotein (LDL) cholesterol. LLT aims to reduce the LDL cholesterol level below 55 mg/dL (1.4 mmol/L) and/or to decrease the LDL cholesterol level by at least 50% [6]. Since 2019, guidelines have recommended reducing LDL cholesterol to even less than 40 mg/dL in very high risk patients, for example, after a second CHD-related event [4].

The cornerstone of LLT is the administration of a maximum dose of statins, with a number of studies demonstrating that high-potency statins (e.g., atorvastatin, rosuvastatin) are superior to low-potency statins (e.g., pravastatin, simvastatin) [7,8,9]. However, some patients do not respond sufficiently to statin monotherapy. Moreover, statin therapy can cause severe adverse effects, such as myotoxicity, which occurs in the form of myopathy, myalgia, myositis, or rhabdomyolysis [10,11]. A recent study associated high doses of statins with an increased risk for osteoporosis [12]. The only available alternatives to statin monotherapy is combination therapy with either ezetimibe or inhibitors of proprotein convertase subtilisin/kexin type 9 (PCSK9) [13,14]. For example, combination therapy with ezetimibe and simvastatin was reported to reduce LDL cholesterol levels, to decrease adverse effects such as nonfatal myocardial infarction and stroke, and to lower the rates of cardiovascular death [15].

Compliance to medication often poses problems because high cholesterol levels go unnoticed, and patients may skip or even stop taking the drug [16,17]. Participation in cardiac rehabilitation (CR), however, leads to better adherence, and to a significant reduction in mortality among CHD patients [18]. Hence, CR is a fundamental component for successful long-term CHD treatment [19,20,21]. Data from almost 100,000 CHD patients enrolled in about 150 randomized trials have demonstrated the benefit of CR on both cardiovascular and total mortality [22,23]. Furthermore, many studies have established that CR improves quality of life as well [24,25]. In Germany, standard of care for CHD patients after ACS or CABG surgery comprises a multimodal 3-week CR at specialized rehabilitation centers [18,24,26,27,28]. This multimodal rehabilitation attempts to both optimize drug therapy and educate patients on the impact and possible adverse effects of drugs in order to increase compliance with drug treatment [18,26,27,28]. Additionally, CR commonly implements intensive programs on five days a week, including psychosocial support, physical exercise, and nutrition counseling [18].

In this analysis of the German multicenter Lipid-Lowering-Therapy-Rehabilitation registry (LLT-R), we focused on the effect of LLT in patients with diabetes mellitus and CHD. We interrogated to what extent CHD patients with diabetes mellitus benefit from LLT during CR.

## 2. Methods

### 2.1. Study Design

The LLT-R registry included 1100 patients who were admitted to one of the six participating German rehabilitation clinics. Inclusion criteria for this study were a minimum age of 18 years, diagnosis of CHD, and enrollment in LLT. The only exclusion criterion was the absence of written informed consent. The ethics committee of the Medical Association of Saxony-Anhalt and the local ethics committees of the participating clinics approved this study (ClinicalTrials.gov Identifier: NCT02749279).

### 2.2. Patient Data

A central database (online-CRF) recorded all relevant baseline parameters, which included indication for rehabilitation, LLT and other drug treatments, all comorbidities, age, sex, BMI, and standard laboratory parameters (e.g., total, LDL, and HDL cholesterol and triglycerides). Moreover, the database contained information on LLT at the beginning of CR, at discharge, as well as on the advice given to general practitioners regarding how LLT should be managed after discharge.

Diabetes was defined as previously diagnosed (under treatment) or newly diagnosed disease during CR according to current guidelines (i.e., HbA1c > 6.5% or 48 mmol/mol, fasting glucose >7 mmol/L, random plasma glucose >11.0 mmol/L or 2 h plasma glucose >11.0 mmol/L in an oral glucose tolerance test) [1]. The study included both type 1 diabetes (approximately 2%) and type 2 diabetes (approximately 98%) patients.

### 2.3. Cardiac Rehabilitation

The cardiac rehabilitation for patients with CHD included diagnostic procedures such as cycle ergometry, echocardiography, and ECG. Patients who were unable to perform cycle ergometry performed a 6 min walking test instead. A sports and rehabilitation program was set up for each patient taking into account physical fitness, severity of primary disease, comorbidities, and other confounding parameters. Patients in good clinical condition participated in heart rate-monitored cycle ergometry training for 30 min. In addition, these patients participated in Nordic walking, medical training therapy, aquatic therapy, and intensive gymnastics or exercise. Patients who were less physically fit participated in group exercises, chair exercises, walking exercises, and personal training sessions. Data on patient assignment according to physical fitness levels were not collected. All patients, irrespective of their physical fitness, attended seminars and lectures over the course of the 3 week rehabilitation program, and received their medication from nurses during their stay.

### 2.4. Follow-Up

During follow-up, patients were contacted by mail 3 and 12 months after discharge to inquire about drug therapy (in particular concerning LLT) and rehospitalization, especially in connection with atherosclerotic diseases, such as recurrent acute coronary syndromes (ACS). Additional information was collected on the rationale and the responsible party for changes in medication. In addition, data on total, LDL, and HDL cholesterol as well as triglyceride levels were collected during follow-up. Patients who failed to return the questionnaires were contacted via telephone to conduct an interview with the patient or his/her relatives. Occasionally, the patient’s physician was contacted as well. Civil registration offices were contacted if this information could not be retrieved from these sources, and information was requested about current addresses or date of death. This study employed a monitoring protocol that was developed by the Coordination Center for Clinical Studies, Martin Luther-University Halle Wittenberg, Germany (KKS Halle).

### 2.5. Statistical Analysis

Continuous variables were described as mean and standard deviation, skewed variables as median and 25% and 75% quartiles. Categorical variables were documented as a percentage. A *t*-test was used to compare metric, normally distributed variables. For skewed variables, the Mann-Whitney U-test was employed. The chi-squared test was used for normally distributed, categorical variables. Odds ratios were calculated via chi-square test from contingency tables. A one-way ANOVA was employed for comparisons between diabetic and nondiabetic patients over time, and for evaluating the time course of lipid levels. A *t*-test was performed for post hoc pairwise comparison. Results were deemed significant for *p*-values lower than 0.05. Statistical analysis was performed with SPSS Statistics (IBM^®^ SPSS^®^ Statistics 25, Chicago, IL, USA).

## 3. Results

### 3.1. Patient Characteristics

Patient characteristics are presented in Table 1. The registry included 76.1% male and 23.9% female patients. Main diagnoses were NSTEMI (31.8%), STEMI (29.6%), and CABG surgery (26.4%). In 369 patients (33.9%), diabetes mellitus was diagnosed. On average, diabetic patients were 3 years older than nondiabetic patients (65.5 ± 9.0 years vs. 62.2 ± 10.9 years, *p* < 0.001). In addition, diabetic patients showed a higher BMI (30.2 ± 5.2 kg/m^2^ vs. 27.8 ± 4.2 kg/m^2^), larger waist circumference (107.8 ± 12.9 cm vs. 101.2 ± 11.6 cm), higher systolic blood pressure (135.6 ± 21.9 mmHg vs. 132 ± 19.9 mmHg, *p* < 0.001), and higher heart rate (75.8 ± 11.6 bpm vs. 71.9 ± 12.4 bpm, *p* < 0.001) than nondiabetic patients. Furthermore, renal function was reduced in diabetic patients (creatinine: 1.1 ± 0.5 mg/dL vs. 1.0 ± 0.3 mg/dL; eGFR: 74.0 ± 22.2 mL/min vs. 78.8 ± 18.0 mL/min, *p* < 0.001 for both), and hemoglobin levels were lower (12.9 ± 1.9 g/dL vs. 13.5 ± 3.7 g/dL, *p* < 0.001). Information on HbA1c, which was available for only 243 diabetics (65.9%), yielded a value of 6.7 ± 0.9% (or 50 ± 10 mmol/mol). Diabetic patients showed three-vessel CHD significantly more often than patients without diabetes (*p* < 0.001; Table 1). 

### 3.2. Low LDL Levels among Diabetic and Nondiabetic Patients

Recent guidelines recommend LDL cholesterol levels <55 mg/dL for LLT [6]. Hence, we evaluated the impact of diabetes mellitus on this particular patient subgroup. The data from this registry showed that patients with diabetes mellitus were more likely to reach this goal by the time of admission to CR than nondiabetic patients (OR 1.9; 95% CI 1.3 to 2.9). 

**Figure 1:**
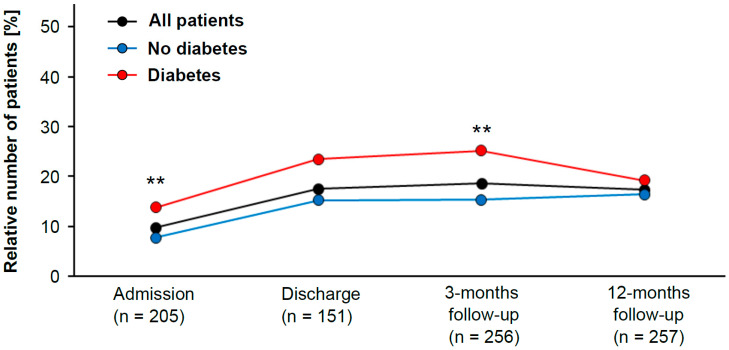
Patient population with low LDL cholesterol levels (classified as <55 mg/dL) during study period (in black). Patients are subdivided into diabetic (red) and nondiabetic (blue) patients. The statistically significant difference between diabetic and nondiabetic patients was evaluated with the Pearson’s chi-squared test (** indicates *p*-value < 0.01).

In addition, the group with LDL cholesterol <55 mg/dL contained significantly more diabetic than nondiabetic patients (chi-squared test, *p* = 0.001) (Figure 1). The same trend was observed at the time of discharge, but the analysis failed to reach statistical significance (chi-squared test, *p* = 0.068). At 3 months after CR, diabetes patients were again more likely to have LDL cholesterol levels <55 mg/dL than those without diabetes (OR 1.9; 95% CI 1.2 to 2.9). Once again, there were significantly more diabetic patients than nondiabetic patients in the group with LDL cholesterol <55 mg/dL (chi-squared test, *p* = 0.006) (Figure 1). After 12 months of follow-up, however, there was no difference between diabetic and nondiabetic patients (chi-squared test, *p* = 0.413).

### 3.3. Lipid Levels of the Patients during CR

**Figure 2:**
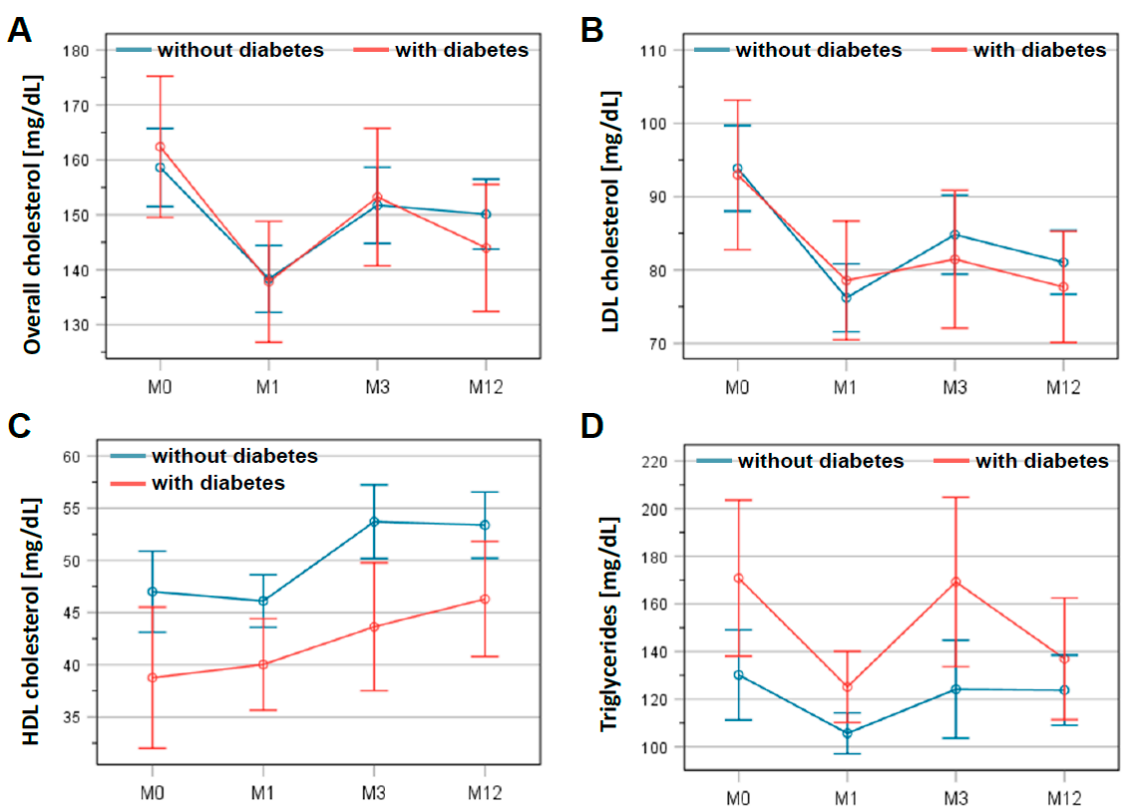
Changes in lipid levels in patients with and without diabetes mellitus during study period. Levels of overall cholesterol (**A**), LDL cholesterol (**B**), HDL cholesterol (**C**), and triglycerides (**D**) are presented at the beginning (M0) and the end of the cardiac rehabilitation (M1) as well as 3 months (M3) and 12 months (M12) after discharge. The graphs show the mean and the error bars represent the 95% confidence interval. Patients with diabetes are indicated in red, patients without in blue.

A time course of lipid levels is presented in Figure 2. Statistical analysis showed a significant reduction in total cholesterol: 156.0 ± 37.5 mg/dL at discharge to 149.6 ± 42.5 mg/dL at 3 months after CR (*p* = 0.002). This lower level was confirmed at the end of follow-up at 12 months (146.2 ± 37.3 mg/dL; *p* < 0.001). Similarly, LDL cholesterol dropped within 3 months from 91.4 ± 30.8 mg/dL at discharge to 81.2 ± 30.2 mg/dL at 3 months after CR (*p* < 0.001), remaining at this level until the end of the follow-up (79.3 ± 27.2 mg/dL; *p* < 0.001). These results did not differ significantly between patients with and without diabetes mellitus (Figure 2A,B). In contrast, HDL cholesterol increased from 45.0 ± 13.8 mg/dL at discharge to 49.5 ± 14.8 mg/dL at 3 months (*p* < 0.001), remaining at this level until 12 months of follow-up (49.5 ± 14.4 mg/dL; *p* < 0.001). Moreover, HDL cholesterol levels differed significantly between patients with and without diabetes mellitus (*p* < 0.01; Figure 2C). For triglycerides, the differences between the two patient groups were significant (*p* = 0.027) (Figure 2D). However, the average triglyceride levels for all patients in this registry were 137.5 ± 77.0 mg/dL at the beginning of CR and remained virtually unchanged at 135.5 ± 78.9 mg/dL on average after 12 months of follow-up.

### 3.4. LLT during Study Period

**Figure 3:**
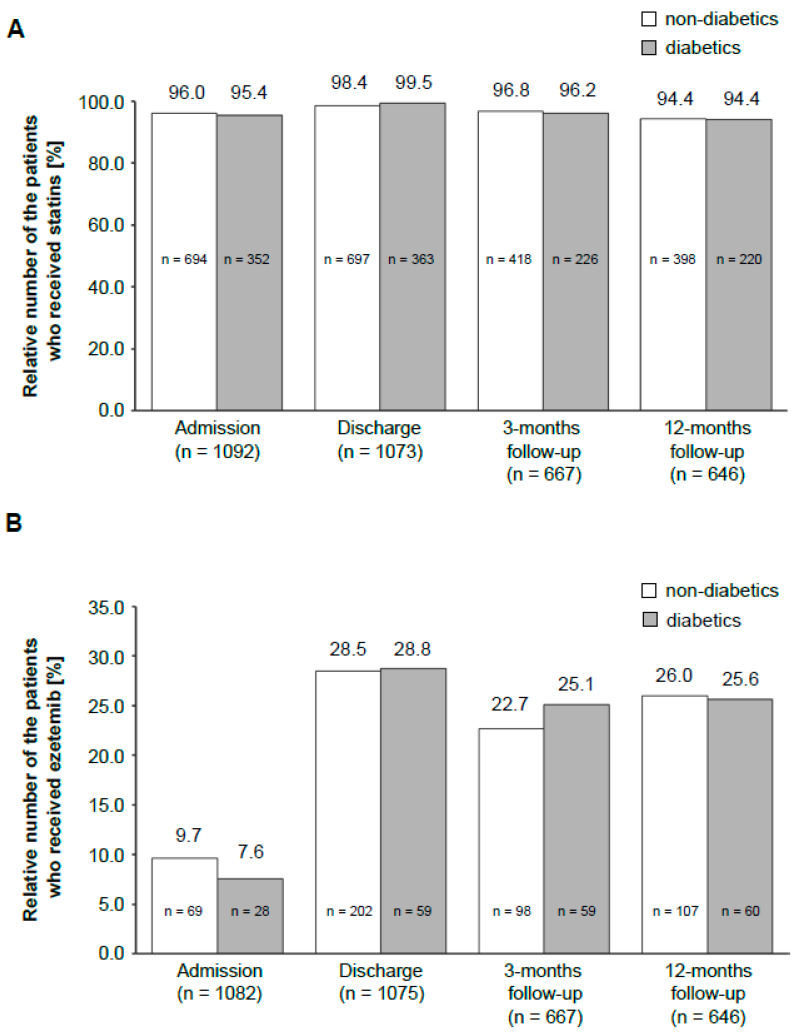
Distribution of LLT drugs in the patient population during the study period. The bar graph shows a comparison of the administration of statins (**A**) and ezetemib (**B**) among diabetic and nondiabetic patients at different time points. The number above the bar graph indicates the percentage of diabetics or nondiabetics, respectively, who received the drug. The number in the bar graph presents the total number of patients. The number on the x-axis shows the total amount of patients in this particular study period.

This registry also assessed the differences in LLT between diabetic and nondiabetic patients. There was no significant difference for statins (Figure 3A) in the two groups. The same was observed for ezetimibe, which is the most often used alternative to statins (Figure 3B).

### 3.5. Drug Therapy

Besides lipid-lowering drugs, concomitant medication was analyzed during CR (see Table 2). The analysis did not show any significant difference between diabetic and nondiabetic patients for oral anticoagulants or platelets inhibitors, with Prasugrel being the only exception: Prasugrel was used less frequently by diabetic than nondiabetic patients (14.8% vs. 27.9%, *p* < 0.001). Regarding antihypertensive drugs, diabetic patients used significantly more diuretics (56.4% vs. 38.1%, *p* < 0.001), angiotensin II receptor blockers (ARB; 37.5% vs. 28.6%, *p* = 0.005), and calcium channel blockers (CCB; 30.4% vs. 18.2%, *p* < 0.001) than patients without diabetes. In contrast, diabetic patients were medicated less frequently with angiotensin-converting enzyme (ACE) inhibitors (57.6% vs. 66.1%) (*p* = 0.007).

### 3.6. Antidiabetic Drugs

In Table 3, antidiabetic medication use is presented. Metformin was the most commonly used antidiabetic drug in 48.0% of the patients at admission and remained the preferred drug through the 12-month follow-up period (51.7% of the patients). DDP-4 inhibitors were also used frequently according to the registry data (26.8% at admission and 26.5% at the end of the study). The use of insulin declined from 33.3% at admission to 26.9% at the end of the documented follow-up. Other antidiabetic drugs, such as sulfonylureas, GLP1 agonists, and meglitinides, were used in less than 5% of the patients at any time. Of note, SGLT2 inhibitor use increased from 3.3% at admission to 7.3% during the 12-month follow-up period.

## 4. Discussion

This prospective, multicenter registry study investigated the effect of LLT on CHD patients in a CR setting in order to assess the benefit of this therapy for diabetics and nondiabetics. The study showed a significant reduction in LDL cholesterol levels for all patients, but the improvement was greater for diabetic patients.

The multicenter LLT-R registry provided a representative cross-section of CHD patients and the treatment situation during and after CR in Germany. The registry data are based on only one exclusion criterion (i.e., the lack of informed consent) and the overall characteristics of the patient cohort, such as an average age of 63 years and less than 25% females [18,24,25,26,27]. Multimodal rehabilitation in Germany results in several improvements during start and end of rehabilitation, including decreases in systolic blood pressure, heart rate, and waist circumference, but not in BMI [18,24]. These findings are in excellent agreement with our observation regarding the unchanged body weight during follow-up after discharge from CR. Furthermore, effective CR could also account for the observed decline in insulin therapy during the course of LLT in the registry.

In agreement with the literature, this registry showed that total and LDL cholesterol levels changed within the first 3 months after CR, and then remained at that level, irrespective of prevalent diabetes mellitus. By contrast, the HDL levels in nondiabetic patients remained constant 3 months after CR, whereas HDL levels in diabetic patients continued to increase in the same period. In addition, our analysis indicated a faster decline in LDL cholesterol levels below 55 mg/dL in diabetes mellitus patients than in patients without diabetes. It should be stated, however, that the few measurement time points in the follow-up period provide only a low resolution of the kinetics of these adjustments. In any case, these observations suggest that both diabetics and nondiabetics benefit from LLT regarding LDL cholesterol levels. In contrast, it is unclear why diabetic patients show a more pronounced increase in HDL cholesterol levels than nondiabetic patients, and thus seemingly benefit even more from LLT. This notion is supported by an earlier study that found a greater risk reduction for major coronary events in diabetic (42%) than in nondiabetic patients (32%) [27]. One explanation could be that antidiabetic drugs influence cholesterol levels in this setting. Another explanation could be differences in metabolic and physiological process between diabetics and nondiabetic patients. A better understanding has implications for the recruitment of diabetic patients to CR, and the role of antidiabetic medication within LLT. Further studies are required to elucidate the biological reason for the differing outcome among both patient groups. 

In the cohort of this CR registry, 33.9% of patients had received a diagnosis of diabetes mellitus (Table 1), whereas the overall rate of diabetic patients among all cardiac events in the German population varies between only 10% and 16% [29]. The overrepresentation of patients with diabetes mellitus in the CR registry cohort is in agreement with the role of diabetes mellitus as a major risk factor for development of CHD [1], hence improving quality of life and prognosis in CHD patients. This is particularly relevant for CHD patients with diabetes mellitus who tend to have increased lipid levels the higher number of high risk patients, such as elderly people who have suffered myocardial infarction and who are admitted more frequently to CR than younger diabetic patients with less severe cardiac events. Nonetheless, this discrepancy between the general population and the registry is a coincidental observation that requires thorough statistical assessment. In any case, the findings presented here have implications for CR patients or patients with recurrent cardiac events. This is particularly important in light of the continuously increasing magnitude of this patient group due to the growing number of multimorbid and older patients.

There are several limitations of this registry. First of all, it is difficult to interpret the data set due to the observational and nonrandomized design of the patient cohort. Changing the design of this study by introducing a control group is almost impossible as every patient in Germany has the litigable right to participate in CR after ACS or CABG [18,24,25,26,27]. Nonetheless, this study aimed for the highest possible data quality. The Coordination Center for Clinical Studies, Martin Luther-University Halle Wittenberg, Germany (KKS Halle), enrolled patients consecutively on a prospective basis in order to provide adequate monitoring and to record all relevant patient information.

In addition, there may be potential incoherencies in LLT medication in this registry. In general, the study showed no differences in treatment of diabetic and nondiabetic patients during LTT. Nonetheless, patients received different drugs during the course of CR in order to address clinical needs, such as duration and severity of disease as well as comorbidities. Consequently, these changes in medication confound the association between treatment and outcome, thus introducing channeling or allocation biases. Unfortunately, compliance was not assessed during the CR, but rehabilitation centers and patients enrolled in prospective studies are more likely to adhere to guideline-oriented therapy than patients outside of such centers or studies. Since nurses provided patients with their medication during the three weeks of CR, it is reasonable to assume that the overwhelming majority of patients adhered to drug therapy.

Moreover, different types of diabetes mellitus could not be addressed separately in this registry as data on the specific types were limited and inconclusive. This would have been an ideal scenario but the authors are aware of the fact that ideal scenarios do not exist in the CR setting, and hence this limitation reflects the reality of the rehabilitation setting. Similarly, this study did not collect data on the fitness level of the patients and their assignment to CR measures. Therefore, it cannot be excluded that allocation of patients to different exercise regimes within the CR framework may have influenced the response to LLT. Lastly, a follow-up period of 12 months is rather short for monitoring lifelong chronic diseases. Hence, a longer follow-up period might have shown further differences between patients with and without diabetes mellitus.

## 5. Conclusions

This study demonstrated that implementation of LLT in context of CR is able to reduce LDL cholesterol within a short time period of 3 months in patients with CHD. The outcome of this prospective study showed that concomitant diabetes mellitus in addition to antidiabetic medication did not impair the efficacy of LLT during CR. On the contrary, patients with diabetes seemed to benefit most from LLT during CR, as they reached LDL treatment goals significantly better than patients without diabetes mellitus during CR.

## Figures and Tables

**Table 1:** Overview of patient characteristics at the end of CR.

Characteristic		All Patients				without Diabetes				with Diabetes				*p*-Value ^a^
		N	%	$\bar{X}$	SD	N	%	$\bar{X}$	SD	N	%	$\bar{X}$	SD	
Age (years)		1087	100	63.4	10.4	716	65.9	62.2	10.9	369	33.9	65.6	9.0	**0.000**
Body Weight (kg)		1083	100	84.9	16.5	714	65.9	83.1	15.5	367	33.9	88.4	17.9	**0.000**
BMI (kg/m²)		1082	100	28.6	4.7	711	65.7	27.8	4.2	369	34.1	30.2	5.2	**0.000**
Waist circumference (cm)		904	100	103.5	12.5	588	65.0	101.2	11.6	315	34.8	107.8	12.9	**0.000**
Systole (mmHg)		1086	100	133.2	20.6	715	65.8	132.0	19.9	369	34.0	135.6	21.9	**0.023**
Diastole (mmHg)		1086	100	78.0	11.4	715	65.8	78.2	11.3	369	34.0	77.7	11.6	0.556
Heart rate (bpm)		1085	100	73.2	12.3	714	65.8	71.9	12.4	369	34.0	75.8	11.6	**0.000**
Creatine [mg/dL]		1084	100	1.0	0.4	713	65.8	1.0	0.3	369	34.0	1.1	0.5	**0.000**
GFR [mL/min]		1083	100	77.1	19.7	712	65.7	78.7	18.0	369	34.1	74.0	22.2	**0.000**
Hemoglobin [g/dL]		1075	100	13.2	5.6	709	66.0	13.5	3.7	366	34.0	12.6	1.9	**0.000**
HBA1c [%]										243		6.7	0.9	n/a
Sex	Male	826	76.1			557	77.8			269	72.9			0.073
	Female	259	23.9			159	22.2			100	27.1			
Indication for admission	NSTEMI	345	31.8			231	32.3			114	30.9			
	STEMI	321	29.6			239	33.4			82	22.2			
	CABG	286	26.4			165	23.0			121	32.8			
	PCI/Stent	61	5.6			35	4.9			26	7.0			
	Valve	37	3.4			25	3.5			12	3.3			
	Others	35	3.2			21	2.9			14	3.8			
Affected blood vessels	1-CAD	289	25.0			203	28.6			65	17.9			**0.000**
	2-CAD	274	25.5			200	28.1			74	20.4			
	3-CAD	532	49.5			308	43.3			224	61.7			

^a^ statistical difference between patients with diabetes and without. Statistical significance is indicated in bold. BMI, body mass index; CABG, coronary artery bypass surgery; CAD, oronary artery disease; GFR, glomerular filtration rate; HbA1c, glycated hemoglobin; NSTEMI, non-ST elevation myocardial infarction; PCI, percutaneous coronary intervention; SD, standard deviation; STEMI, ST elevation myocardial infarction; $\bar{X}$, sample mean.

**Table 2:** Comparison of diabetic vs. nondiabetic patients at baseline during non-LLT drug therapy.

Characteristic		All Patients		without Diabetes		with Diabetes		*p*-Value ^a^
		N	%	N	%	N	%	
Platelet inhibitors	ASA	1031	97.6	680	97.8	351	97.2	0.535
	Clopidogrel	251	25.8	151	24.0	100	29.2	0.080
	Prasugrel	224	23.3	174	27.9	50	14.8	**0.000**
	Ticagrelor	342	34.4	232	35.9	110	31.6	0.178
Oral anticoagulants	Vitamin K antagonist	64	33.7	39	32.2	25	36.2	0.575
	Dabigatran	52	28.0	28	23.9	24	34.8	0.111
	Rivaroxaban	5	2.8	2	1.8	3	4.5	0.363
	Edoxaban	6	3.4	6	5.4	0	0.0	0.085
	Apixaban	25	13.7	21	18.3	4	6.0	0.020
Anti-hypertensives	Diuretics	446	44.5	249	38.1	197	56.4	**0.000**
	ACE inhibitors	654	63.2	449	66.1	205	57.6	**0.007**
	ARB	307	31.7	180	28.6	127	37.5	**0.005**
	Renin inhibitors	3	0.3	3	0.5	0	0.0	0.556
	CCB	221	22.5	117	18.2	104	30.4	**0.000**
	Beta blocker	959	90.4	632	90.5	327	90.1	0.809
	MRAs	121	12.5	84	13.4	37	10.9	0.281

^a^ statistical difference between patients with diabetes and without. Statistical significance is indicated in bold. ASA, acetylsalicylic acid; ACE, angiotensin-converting enzyme; ARB, angiotensin II receptor blocker; CCB, calcium channel blocker; MRA, mineralocorticoid receptor antagonist.

**Table 3:** Antidiabetic drugs among patients with diabetes mellitus during study period.

Antidiabetics	Admission		Demission		3-Months Follow-Up		12-Months Follow-Up	
	n = 369		n = 364		n = 235		n = 234	
	N	%	N	%	N	%	N	%
Any antidiabetics	272	73.7	265	72.8	165	70.2	164	70.1
Metformin	177	48.0	194	53.3	126	53.6	121	51.7
Sulfonylureas	18	4.9	6	1.6	6	2.6	8	3.4
DPP-4 inhibitors	99	26.8	103	28.3	57	24.3	62	26.5
GLP1 agonists	7	1.9	9	2.5	3	1.3	7	3.0
SGTL2 inhibitors	12	3.3	21	5.8	16	6.8	17	7.3
Meglitinides	2	0.5	2	0.5	3	1.3	2	0.9
Insulin	123	33.3	108	29.7	56	23.8	63	26.9

## Data Availability

All source data are available upon request.
